# Is Harmonicity a Misnomer for Cultural Familiarity in Consonance Preferences?

**DOI:** 10.3389/fpsyg.2022.802385

**Published:** 2022-01-28

**Authors:** Imre Lahdelma, Tuomas Eerola, James Armitage

**Affiliations:** Department of Music, Durham University, Durham, United Kingdom

**Keywords:** consonance, dissonance, harmonicity, roughness, tonality, psychoacoustics, familiarity

## 1. Introduction

The question of simultaneous consonance and dissonance—the relative agreeableness/stability vs. disagreeableness/instability of pitch combinations—has received a substantial amount of scholarly attention recently (see e.g., Friedman et al., 2021; Harrison, 2021; Lahdelma et al., 2021). A consensus has been emerging in recent years that the Western notion of consonance/dissonance (C/D) is a combination of the acoustic phenomena of *roughness* and *harmonicity*, and the cultural effect of *familiarity* (see e.g., Harrison and Pearce, 2020). Roughness denotes the sound quality that arises from the beating of frequency components, harmonicity in turn how closely a sonority's spectrum corresponds to a harmonic series. Familiarity denotes the prevalence of sonorities in a given musical culture which affects how familiar listeners are with different pitch combinations present in actual music (see Johnson-Laird et al., 2012; Harrison and Pearce, 2020; Lahdelma and Eerola, 2020). In the light of recent research spanning cross-cultural fieldwork and psychoacoustic approaches we argue that the presumably acoustic (bottom-up) contribution of harmonicity is in fact so closely related to cultural familiarity (top-down) that its unique role as a predictor of consonance preferences can be contested. We propose that the role of ‘harmonicity’ may in fact be familiarity with the tonal framework that all Western listeners know either explicitly or implicitly (Johnson-Laird et al., 2012).

## 2. Harmonicity Reflects Acquired Aspects of Consonance Preferences

Both roughness and harmonicity are considered 'natural' components of C/D because of their apparent perceptual universality (Parncutt and Hair, 2011) as opposed to the cultural component of familiarity. Somewhat paradoxically it has been reported that musicians are more sensitive to harmonicity than non-musicians (McDermott et al., 2010; Smit et al., 2019). Smit et al. (2019) found that chords with higher levels of harmonicity are perceived as more consonant and that this effect is stronger for musically more sophisticated participants than it is for musically less sophisticated participants. Conversely, Smit et al. (2019) observed that the effect of roughness on the perception of C/D in unfamiliar chords was not moderated by musical sophistication. This is especially curious in the light that empirical data about automatic reactions to consonance/dissonance have not found differences with regard to musical sophistication when measured with neural responses (Linnavalli et al., 2020) or with a reaction time task (Lahdelma et al., 2020; Armitage et al., 2021) as opposed to self-reports (see e.g., Lahdelma and Eerola, 2016; Smit et al., 2019; Linnavalli et al., 2020). Causal explanations for why harmonicity's effect in perceived C/D is moderated by musical training have remained speculative—it has been suggested that musical experience amplifies preferences for harmonicity (McDermott et al., 2010) or that people who are sensitive to harmonicity are drawn to music and hence have higher musical sophistication (Smit et al., 2019). Furthermore, it has also been demonstrated that in the condition of amusia (i.e., a neurogenetic disorder characterized by an inability to recognize or reproduce musical tones) there is no preference for harmonicity over inharmonicity, while the aversion to roughness remains similar to that of the general population (Cousineau et al., 2012). On a related note, recent research has demonstrated the strong role of learning (Weiss et al., 2020) and cultural familiarity (Lahdelma and Eerola, 2020) in the consonance preferences of Western listeners. In the study by Weiss et al. (2020) the preferences for consonance in musical intervals increased with age and were predicted by changing preferences for harmonicity, while in the study by Lahdelma and Eerola (2020) the correlation between consonance and preference in chords were dependent on familiarity for both musicians and non-musicians. These findings point to a culturally acquired rather than an inherent acoustic (harmonicity) aspect of consonance preferences, although it is important to note that these aspects are likely to interact and form a continuum rather than a sharp dichotomy; human cognition is shaped by a dynamic and ever changing interaction with the environment.

Cross-cultural research offers further insight into this process of learning in terms of consonance/dissonance preferences. McDermott et al. (2016) investigated how C/D is perceived among the Tsimané, an indigenous population living in the Amazon rainforest (Bolivia) with limited exposure to Western culture. They found that the Tsimané showed no preference for consonance (harmonic intervals and chords) but did show an aversion to roughness (in small musical intervals) when the stimuli were presented diotically (simultaneous presentation to each ear) as opposed to separate ears (dichotically). This finding is notably in line with a recent psychoacoustic experiment conducted on Western listeners demonstrating that intervals within the critical bandwidth (minor and major seconds) elicit quicker automatic negative responses compared to consonant intervals; in other words, contrasts in roughness but not in harmonicity drive automatic affective responses to consonant/dissonant musical intervals (Armitage et al., 2021). Conversely, McDermott et al. (2016) found that Western listeners had an aversion to dissonant intervals (the minor second, major second, and tritone) in both the diotic and dichotic conditions. This is highly interesting in the light that the beating effects resulting in perceived roughness are considerably stronger when two tones are presented diotically rather than dichotically (see Grose et al., 2012; Harrison and Pearce, 2020); the interference of intervals can be essentially eliminated by dichotic presentation (Harrison and Pearce, 2020). Conversely, harmonicity detection is thought to be a central process that combines information from both ears (Houtsma and Goldstein, 1972) and should thus be unaffected by dichotic presentation. As the Tsimané had an aversion only to roughness and not inharmonicity (unlike the Western listeners who were sensitive to both) these findings imply that what has been labeled as ‘harmonicity’ predicting consonance preferences is in fact shaped by learning and cultural familiarity (see also Lahdelma and Eerola, 2020; Weiss et al., 2020). Another recent cross-cultural research endeavor comparing the perception of chords across non-Western (two remote Northwest Pakistani tribes with limited exposure to Western music) and Western listeners echoes the findings of McDermott et al. (2016). The Northwest Pakistani tribes did not indicate any preference for the consonant major triad but had a clear aversion to the highly dissonant chromatic cluster chord (Lahdelma et al., 2021).

Finally, a recent study using culturally unfamiliar stimuli on Western participants in the form of an alternative tuning system (Bohlen–Pierce chromatic just intonation tuning scale) also concluded that harmonicity and consonance are not significantly related across all possible intervals and trichords (Friedman et al., 2021), although Smit et al. (2019) on the other hand found using the same tuning system that harmonicity correlates positively with pleasantness ratings. However, as is evident from the report by Friedman et al. (2021) and from a later re-analysis of the said study's data by Bowling (2021), the problem with the Bohlen-Pierce scale is that familiarity with this tuning system is hardly binary but rather a continuum as listeners have been shown to tolerate rather large (between 20 and 45 cents) deviations from equal temperament (see Zatorre and Halpern, 1979; Rakowski, 1990). Trying to get around familiarity issues with unconventional tunings easily leads to a problem of discarding large chunks of data (see Bowling, 2021; for a critique see Goffinet, 2018). Bypassing cultural familiarity with alternative tunings when targeting Western listeners is evidently borderline impossible, a case in point being the fact that Western listeners can easily adjust to deviations from equal temperament, for example, when listening to historical tunings.

## 3. Is the Role of ‘Harmonicity’ in Consonance Preferences Actually Familiarity With Tonality?

Following from these observations we propose that what has been identified as the role of ‘harmonicity’ in previous research on C/D preferences might in fact be a knowledge of tonal relations. According to Johnson-Laird et al. (2012) the relevant principles of tonality are tacitly represented in the minds of all Western listeners—in other words, the typical context of pitch combinations is known explicitly by musicians and to a lesser degree implicitly by non-musicians. Based on empirical findings, Johnson-Laird et al. (2012) propose the concept of *tonal consonance/dissonance* to explain cultural familiarity's role in C/D preferences. According to this concept the C/D of isolated pitch combinations depend on the scales in which they occur: pitch combinations occurring in a major scale are less dissonant than pitch combinations occurring only in a minor scale, which in turn are less dissonant than pitch combinations occurring in neither sort of scale. Eerola and Lahdelma (2021) took the parsimony of the tonal consonance/dissonance idea by Johnson-Laird et al. (2012) a step further still by collapsing minor and other scales together to create a simple implementation called the *Tonal Dissonance Model*, which assesses whether a pitch combination can be constructed from a major scale (1) or not (0). Their choice was motivated by analyzing the contribution of the three principles of the original model by testing each principle as a binary coded variable in regression to predict consonance ratings together with roughness, familiarity, and spectral envelope predictors (see Eerola and Lahdelma, 2021). Strikingly, Eerola and Lahdelma (2021) found this simple binary division model functions like a harmonicity model; it is remarkable how complex models calculating harmonicity of the partials do not perform better than a model that merely checks whether pitch combinations can be created from a diatonic major scale (i.e., whether the pitch combination can theoretically be part of a diatonic major key tonality). It has been demonstrated that musicians tend to perceive specifically culturally familiar chords as more consonant compared to non-musicians (McLachlan et al., 2013; Lahdelma and Eerola, 2016) as per the mere exposure effect (Zajonc, 2001)—this would readily explain the results of previous experiments with regard to the training-based differences in sensitivity to “harmonicity”.

An important question which ostensibly poses a chicken or the egg dilemma for cultural accounts of consonance preferences (see Bowling, 2021) is why some intervals are more attractive than others to become prevalent in tonality to start with. Animal studies that could shed light on the innateness of consonance perception have been inconclusive so far, which is no surprise given the challenges in conducting such studies rigorously. Animal studies can be divided into two main categories: *discrimination studies* and *preference studies* (Toro and Crespo-Bojorque, 2017). There is tentative evidence supporting the notion that diverse non-human species can both discriminate consonance and dissonance (Hulse et al., 1995; Izumi, 2000) and that they may prefer consonance over dissonance (Sugimoto et al., 2010; Chiandetti and Vallortigara, 2011), but also contrasting results have been reported (Brooks and Cook, 2010; Koda et al., 2013; Crespo-Bojorque and Toro, 2015). Although these discrepancies are most likely due to small stimulus sets, small sample sizes, and a lack of replication in such studies (Harrison and Pearce, 2020), this research trajectory is promising and may cast further light on the nature/nurture elements in consonance perception. What is clear, however, is that common scales, including the major diatonic scale, tend to maximize harmonic pitch combinations (see Huron, 1994), although this could alternatively be interpreted as a form of minimizing roughness. Either way, there is evidence suggesting that humans may have a biological predisposition for harmonicity sensitivity (see e.g., Lewis et al., 2009; Wang, 2013; Feng and Wang, 2017) and it has been suggested that this is due to harmonicity being an important hallmark of distinguishing animal vocalizations from other environmental sounds (Bowling et al., 2018). The aversion to roughness would also be in line with this vocal similarity theory, as for example screams (Schwartz et al., 2020) and infant cries (Koutseff et al., 2018) are acoustically rough and hence confer an evolutionary advantage—in addition to the biological substrate to its aversion due to interference in the inner ear (see Jülicher et al., 2001). We propose that while harmonicity does not directly influence C/D preferences (as opposed to roughness, see e.g., Lahdelma et al., 2020; Armitage et al., 2021) it is plausible that it has shaped Western tonality (and is of course an important element in pitch and timbre perception) through the phenomenon of *fusion* which in effect is a *consequence* of harmonicity (McPherson et al., 2020). Stumpf (1890) explains fusion as a tendency for simultaneous sounds to blend perceptually or to be perceived as one sound, and fusion has been put forward as an explanation for some common musical observations relating to common-practice tonality, for example, the prevalence of the major triad by comparison to the minor in spite of their similar roughness, and the prevalence of the dominant seventh chord—musical chords are prevalent if their tones fuse so that many tones are heard as one (Parncutt et al., 2019). Stumpf's views about consonance having a link to fusion have received corroboration from empirical studies (Guernsey, 1928; DeWitt and Crowder, 1987), although more recently McLachlan et al. (2013) found that listeners succeeded in isolating more clearly the components of consonant than dissonant chords, and therefore, contrary to Stumpf's claims, consonant chords were in fact perceived as less fused.

However, this possibly hard-wired harmonicity sensitivity does not necessarily entail aesthetic preference *per se*, and previous research implies that the processing of the auditory system does not rigidly determine the higher cognitive processes of preference choices in terms of consonance perception (see Linnavalli et al., 2020). Moreover, it is vitally important to distinguish inherent attractiveness from harmonicity-induced fusion. McPherson et al. (2020) found that across both Western and non-Western listeners (the Tsimané) perceived fusion was greater for the octave, fifth, and fourth than for the dissonant intervals closest in size. Strikingly, fusion did not predict preferences in Tsimané participants, who did not prefer consonant to dissonant intervals, instead showing a slight preference for larger intervals (McPherson et al., 2020). McPherson et al. (2020) remind that even in Westerners, consonance preferences are not fully predicted by fusion as a consequence of harmonicity and that consonance preferences are evidently subject to some other (presumably culture-specific) influence. An interesting case-in-point here is the history of the major third interval which is highly harmonic but became consonant only over time in Western music (see Hindemith, 1942; Tenney, 1988), familiarity (through frequency of occurrence) evidently driving its perceived consonance instead of an inherent acoustic ‘harmonicity’ effect. This latter observation is in line with cross-cultural research demonstrating a lack of preference for major chords (McDermott et al., 2016; Lahdelma et al., 2021) and major-key chord sequences (Athanasopoulos et al., 2021) in non-Western populations. As this recent cross-cultural research evidence suggests that harmonicity preferences may be restricted to the Western musical culture, it is possible that such preferences are learned both on an individual (Weiss et al., 2020) and on a cultural level (see Lahdelma and Eerola, 2020) through exposure. This preference for harmonicity would arguably arise through exposure to specifically polyphony in a given musical culture (cf. the lack of polyphony and lack of preference for consonance among the Tsimané, see McDermott et al., 2016), although notably Athanasopoulos et al. (2021) and Lahdelma et al. (2021) did not find a preference for highly harmonic background harmonisations and chords among two remote Pakistani tribes with minimal exposure to Western music who nonetheless do have some polyphony in their own music. We propose that future research should address exactly how and why the seemingly universal perception of fusion becomes associated specifically with positive valence and preference for Western listeners (see also Weiss et al., 2020). Also, it is important to keep in mind that in cross-cultural research scholars have to resort to valence-based terminology (pleasantness, preference) as the terms ‘consonance’ and ‘dissonance’ are of course exclusively Western concepts; this terminology simplification has been demonstrated to be problematic also in the context of Western listeners (see Lahdelma and Eerola, 2020) and should be bypassed by coming up with methods that minimize semantic limitations (see e.g., Linnavalli et al., 2020; Armitage et al., 2021).

## 4. Conclusions

We have argued in the light of cumulative research data spanning cross-cultural and psychoacoustic experiments that the presumably acoustic (bottom-up) role of harmonicity in consonance preferences is in fact knowledge of the Western tonal framework and is hence a misnomer for a cultural (top-down) effect. We agree with the conclusion drawn by McDermott et al. (2016) according to which consonance preferences are not innate or universal (cf. Bowling et al., 2018) and seem to depend on exposure to particular types of music, presumably those that feature consonant harmony. However, we propose taking this line of thinking one step further and argue that it is not just familiarity with consonant harmony but with specifically the framework of tonality that both musicians and (to a lesser degree) non-musicians are familiar with in the West (Johnson-Laird et al., 2012). In other words, it is possible that what in previous research has been identified as musicians' higher sensitivity to harmonicity (see McDermott et al., 2010; Smit et al., 2019) is actually sensitivity to whether pitch combinations are familiar from the most common (major) tonality. Hence, we argue that harmonicity is not a direct predictor of consonance preferences but that it has presumably shaped Western tonality through fusion. Beyond this the consonance preferences in Western music have presumably been instated by exposure and this finding is in line with cross-cultural research demonstrating a lack of preference for harmonic pitch combinations among non-Western populations (McDermott et al., 2010; Athanasopoulos et al., 2021; Lahdelma et al., 2021). If the revision of harmonicity's role in consonance/dissonance preferences is indeed firmly grounded, we may have come full circle in identifying the key components predicting these preferences: Helmholtz (1875) already drew the conclusion that consonance/dissonance is dependent on a psychoacoustic (roughness) and on a cultural factor (tonality).

## Author Contributions

IL, TE, and JA conceived the article. IL wrote the manuscript. All authors contributed to the article and approved the submitted version.

## Funding

This research was carried out with a grant from the Ella and Georg Ehrnrooth Foundation awarded to IL.

## Conflict of Interest

The authors declare that the research was conducted in the absence of any commercial or financial relationships that could be construed as a potential conflict of interest.

## Publisher's Note

All claims expressed in this article are solely those of the authors and do not necessarily represent those of their affiliated organizations, or those of the publisher, the editors and the reviewers. Any product that may be evaluated in this article, or claim that may be made by its manufacturer, is not guaranteed or endorsed by the publisher.
